# Mast Cell Subsets and Their Functional Modulation by the *Acanthocheilonema viteae* Product ES-62

**DOI:** 10.1155/2013/961268

**Published:** 2013-02-07

**Authors:** Dimity H. Ball, Hwee Kee Tay, Kara S. Bell, Michelle L. Coates, Lamyaa Al-Riyami, Justyna Rzepecka, William Harnett, Margaret M. Harnett

**Affiliations:** ^1^Centre for Immunobiology, Glasgow Biomedical Research Centre, Institute of Infection, Immunity and Inflammation, College of Medical, Veterinary and Life Sciences, University of Glasgow, Glasgow G12 8TA, UK; ^2^Strathclyde Institute of Pharmacy and Biomedical Sciences, University of Strathclyde, Glasgow G4 0RE, UK

## Abstract

ES-62, an immunomodulator secreted by filarial nematodes, exhibits therapeutic potential in mouse models of allergic inflammation, at least in part by inducing the desensitisation of Fc**ε**RI-mediated mast cell responses. However, in addition to their pathogenic roles in allergic and autoimmune diseases, mast cells are important in fighting infection, wound healing, and resolving inflammation, reflecting that mast cells exhibit a phenotypic and functional plasticity. We have therefore characterised the differential functional responses to antigen (via Fc**ε**RI) and LPS and their modulation by ES-62 of the mature peritoneal-derived mast cells (PDMC; serosal) and those of the connective tissue-like mast cells (CTMC) and the mucosal-like mast cells derived from bone marrow progenitors (BMMC) as a first step to produce disease tissue-targeted therapeutics based on ES-62 action. All three mast cell populations were rendered hyporesponsive by ES-62 and whilst the mechanisms underlying such desensitisation have not been fully delineated, they reflect a downregulation of calcium and PKC**α** signalling. ES-62 also downregulated MyD88 and PKC**δ** in mucosal-type BMMC but not PDMC, the additional signals targeted in mucosal-type BMMC likely reflecting that these cells respond to antigen and LPS by degranulation and cytokine secretion whereas PDMC predominantly respond in a degranulation-based manner.

## 1. Introduction

Mast cells are increasingly recognised as playing an important pathogenic role in a variety of allergic and autoimmune diseases [1–4]. However, there is also developing evidence of their participation in tissue repair and resolution of inflammation [5–7], as well as for their exhibiting pathogenic and protective roles in cancer [8, 9]. Such contradictory evidence relating to mast cell function most likely reflects that mast cells, which are haematopoietic cells found in all vascularised organs [10–13], constitute a heterogeneous cell population [14] varying in morphology, function, and location with subpopulations being characterised by their differential protease, eicosanoid, and proteoglycan content [10, 13–17]. Such heterogeneity arises because bone marrow-derived mast cell progenitors [18, 19] arrive in tissue before they are fully matured [20] allowing the different cytokines, hormones, and reactive oxygen and/or nitrogen species produced by various microenvironments to essentially create “custom-made” site-specific mast cells [12–14]. 

Moreover, the functional response of mast cells depends on the stimuli received [12]; for example, following classical activation via the IgE receptor, Fc*ε*R1, mast cells degranulate rapidly (within minutes) to exocytose prostaglandins and leukotrienes as well as preformed cytokines, tryptase, histamine, heparin, and platelet activating factor (PAF) whilst *de novo* synthesised cytokines exhibit a more delayed (hours) release [8, 21]. However, mast cells can also be activated independently of Fc*ε*RI and this can be initiated by cytokines or other proinflammatory mediators [21] reflecting a direct interaction with triggering factors such as LPS, parasite molecules, or allergic stimuli in the skin or the mucosa. Hence, mast cells are frequently the first cell type to respond during inflammation [6], as evidenced by the important roles played by mast cells in bacterial and parasitic infections [22–25]. However, mast cells are also able to influence disease progress subsequently; both directly via the release of proinflammatory mediators, and indirectly via their effects on other immune cells, including dendritic cells (DC), T and B cells, and macrophages [6].

Isolation of *in vivo* differentiated mast cell subsets is difficult due to their limited numbers in tissue [5, 26] and this has led to the development of *in vitro* culture protocols to generate human and mouse mast cells, as defined by their expression of CD117, Fc*ε*R1 and the IL-1 receptor family member, ST2 (suppression of tumorigenicity 2), that can be subclassified as distinct phenotypes due to their differential granular phenotypic and functional responses [14]. Thus, although mast cells make up <5% of the peritoneal cell population, they can be expanded by *in vitro *culture with SCF to create large numbers of homogenous peritoneal-derived mast cells (PDMC) that are serosal-type mast cells and retain most of the morphological, phenotypic, and functional features of mature mast cells [5, 14]. Such PDMC exhibit differential functional responses (generating less lipid mediators, chemokines, and cytokines but displaying stronger degranulation responses) [5, 14] to bone marrow-derived mast cells (BMMC), generated by culture in the presence of IL-3 and SCF [27–29] and often used to represent mucosal-type mast cells (mucosal-type BMMC) [10, 14]. Phenotypically, these BMMC, more closely resemble immature cells and have no identifiable physiological equivalent in tissues [5, 12]. Nevertheless, such BMMC can repopulate both the mucosal and serosal mast cell compartments when adoptively transferred to mast cell-deficient mice, consistent with the proposal that these cells represent precursor cells that require additional site-specific signals to develop into mature tissue mast cells [12, 14]. By contrast, coculture of BMMC with fibroblasts [30, 31] generates connective tissue mast cells (CTMC) which have been used to represent serosal mast cells [10, 14]; more recently it has been shown that these can be differentiated from bone marrow precursors using SCF and IL-4 [14, 32] and CTMC have been implicated as being involved in both autoimmunity [33] and contact hypersensitivity [34].

The phosphorylcholine (PC)-containing excretory-secretory filarial nematode product, ES-62 exhibits broad anti-inflammatory properties including the desensitisation of Fc*ε*RI-mediated mast cell responses and displays therapeutic potential in associated mucosal allergic inflammatory disorders such as asthma [35, 36]. As ES-62 is also protective in autoimmune and allergic connective tissue inflammatory pathologies such as arthritis and contact hypersensitivity, respectively [35, 36], we have therefore investigated its effects on mature PDMC and also on both CTMC and mucosal-type BMMC function in order to better understand mast cell biology as a first step to producing disease tissue-targeted therapeutics based on ES-62 action.

## 2. Materials and Methods

### 2.1. Mice and Reagents

BALB/c and C57BL/6 mice were purchased from Harlan Olac and maintained at the Universities of Glasgow and Strathclyde. All procedures were conducted in accordance with Home Office, U.K. animal guidelines and with the approval of the local ethical committees. Unless otherwise stated, all reagents were obtained from Sigma. 

### 2.2. Peritoneal Derived Mast Cells (PDMC)

PDMC were expanded as described previously [5]. Briefly, cells were harvested following washing of the peritoneal cavity of 6–8 week-old mice with 5 mL sterile, cold RPMI 1640 by centrifugation at 400 g for 5 min and then resuspended in fresh complete RPMI (RPMI with 10% FBS, 4 mM L-glutamine, 100 U/mL penicillin, 100 *μ*g/mL streptomycin, 1 mM sodium pyruvate, 100 *μ*M nonessential amino acids, and 50 *μ*M *β*-mercaptoethanol; Invitrogen Life Technologies) before being incubated at 5 × 10^6^ cell/mL for 2 h at 37°C in tissue culture-treated petri dishes (Corning) to remove adherent cells. The suspension cells were cultured at 0.3 × 10^6^/mL in complete RPMI supplemented with 10 ng/mL recombinant SCF (Pepro Tech) or 4% conditioned medium from the SCF-secreting cell line KLS-C. KLS-C is a CHO (Chinese Hamster Ovary) cell line that produces SCF and was a kind gift from Dr Xiaoping Zhong, Duke University Medical Center. KLS-C cells were cultured at 37°C in Minimum Essential Medium alpha (MEM*α*) without nucleosides (Invitrogen Life Technologies) and with 10% FBS, 100 U/mL penicillin, 100 *μ*g/mL streptomycin and 2.5 *μ*M methotrexate. SCF-enriched supernatant was filtered to remove cell debris and the concentration determined by ELISA and then adjusted with PBS to 550 ng/mL to be used at a final concentration of 22 ng/mL for PDMC. 

### 2.3. Bone Marrow-Derived Mast Cells

Intact femurs and tibias were dissected from BALB/c or C57BL/6 mice and single cell suspensions were obtained by passing bone marrow cells through a 100 *μ*M nylon monofilament gauze (Cadisch Precision Meshes) and lysis of red blood cells for 1 min at 22°C, followed by washing in PBS at 400 g.

Mucosal-type BMMC were derived by culture of bone marrow progenitors [27–29] at 0.5 × 10^6^/mL in RPMI with 10% FBS, 2 mM L-glutamine, 100 U/mL penicillin, 100 *μ*g/mL streptomycin, 1 mM sodium pyruvate, 10 mM HEPES, and 50 *μ*M *β*-mercaptoethanol supplemented with conditioned medium from KLS-C (1%; SCF) and TOP3 (3%; IL-3) cell lines. TOP3 is a cell line that produces IL-3 and was a kind gift from Dr Massimo Gadina, NIH. TOP3 cells were cultured at 37°C in RPMI with 5% FBS, 2 mM L-glutamine, 100 U/mL penicillin, 100 *μ*g/mL streptomycin, 1 mM sodium pyruvate, and 50 *μ*M *β*-mercaptoethanol and 0.4 mg/mL G418. IL-3-enriched supernatant was filtered to remove cell debris and the concentration determined by ELISA, with the stock concentration adjusted with PBS to 1300 ng/mL to be used at a final concentration of 39 ng/mL. Alternatively, mucosal-type BMMC were derived using 10 ng/mL recombinant SCF and 10 ng/mL recombinant IL-3 (Pepro Tech).

CTMC were derived by culture of bone marrow progenitors [14, 32] at 0.8 × 10^6^/mL in RPMI with 10% FBS, 4 mM L-glutamine, 100 U/mL penicillin, 100 *μ*g/mL streptomycin, 1 mM sodium pyruvate, 100 *μ*M nonessential amino acids and 50 *μ*M *β*-mercaptoethanol supplemented with 1% KLS-C conditioned medium (or 10 ng/mL recombinant SCF) and 1 ng/mL recombinant murine IL-4 (Pepro Tech). 

PDMC, mucosal-type BMMC, and CTMC were cultured at 37°C/5% CO_2_ in tissue culture-treated flasks (Greiner Bio-one) for at least 28 days, with adherent cells being discarded. A purity of >95% mast cells was routinely obtained as evidenced by the surface expression of CD117, Fc*ε*R1 and ST2 and viability was determined by Trypan Blue staining. 

### 2.4. Mast Cell Stimulation

Unless otherwise stated, mast cells were sensitised with 0.5 *μ*g/mL murine anti-DNP IgE for 18 h prior to stimulation. In experiments investigating immunomodulation by ES-62, mast cells were incubated with ES-62 (2 *μ*g/mL) simultaneously with IgE during the sensitisation period. Cells were then stimulated (1 × 10^6^ cells/mL except where indicated) by addition of medium, 0.5 *μ*g/mL DNP-HSA to cross-link Fc*ε*R1, 0.5 *μ*g/mL LPS (*Salmonella minnesota*) or PMA (phorbol myristate acetate; 1 *μ*M) plus ionomycin (1 *μ*M). Reactions were terminated after the desired culture period by centrifugation at 400 g and supernatants aspirated for determination of mediator release whilst the cell pellets were stored at −20°C until subjected to Western Blot analysis.

### 2.5. Preparation of Endotoxin-Free ES-62

ES-62 was purified to homogeneity from spent culture medium of adult *Acanthocheilonema viteae* using endotoxin-free reagents as described previously [37]. The purity and identity of each batch were confirmed by SDS-PAGE and the level of endotoxin in the ES-62 sample was determined using a Limulus Amebocyte Lysate (LAL) QCL-1000 kit (Lonza Biologics). ES-62 is used at a working concentration that has an endotoxin reading of <0.003 endotoxin units/mL [37].

### 2.6. Mast Cell Phenotyping

To identify mast cells by positive staining of heparin with Toluidine Blue, cells (0.01 × 10^6^) were cytofuged using a Shandon Cytospin3 (Thermo Shandon) at 500 rpm for 5 min. Slides were air dried before staining with 0.5% Toluidine Blue in 0.5 M HCl for 10–15 min. Images were obtained using an Olympus BX41TF microscope.

Mast cells were also phenotyped by flow cytometric analysis of lineage markers. Briefly, cells were pre-incubated with 50 *μ*L Fc receptor (FcR) blocking buffer (anti-CD16/32, clone 2.4G2, hybridoma supernatant, 10% mouse serum, and 0.1% sodium azide) for 20 min at 4°C prior to incubation with the appropriate flurochrome-conjugated or biotinylated antibodies (2 *μ*g/mL; suspended in 50 *μ*L Fc Block; CD117, eBioscience; Fc*ε*RI, eBioscience; ST2, MD Bioproducts; and TLR4/MD2, eBioscience) for 30 min, 4°C. Following washing, for biotinylated primary antibodies, flurochrome-conjugated streptavidin was added for a further 30 min at 4°C. After labelling, cells were washed twice with 3 mL FACS buffer (PBS containing 2% FBS and 2 mM EDTA) at 1500 rpm for 6 min, 4°C and then resuspended in FACS buffer. To enable exclusion of dead cells from the analyses, cells were either stained with Live/Dead Viability/Cytotoxicity Kit (Invitrogen) before commencement of staining or by the addition of 1 *μ*L 7-AAD (7-Amino Actinomycin D; eBioscience) immediately prior to data acquisition. Cellular fluorescence data were acquired using a Becton Dickinson LSR II or FACSCalibur flow cytometer and analysed using FlowJo software (Tree Star Inc). Analysis was performed on a minimum of 10,000 events.

### 2.7. Calcium Mobilisation

Cells were loaded with the fluorescent calcium-sensing dye Fura-2/AM (5 *μ*M; Invitrogen) in HBSS (145 mM NaCl, 5 mM KCl, 1 mM MgSO_4_, 1 mM CaCl_2_, and 10 mM HEPES) supplemented with 0.18% (w/v) D-glucose and 0.2% (w/v) BSA for 30 min at 37°C in the dark. For measurement of intracellular calcium mobilisation in the absence of extracellular calcium, calcium-free HBSS supplemented with 100 *μ*M EGTA (ethylene glycol tetraacetic acid) to chelate any remaining free calcium, was used. Cells (10^6^) were added to a stirred glass cuvette in a Hitachi F-700 fluorescence spectrophotometer at 37°C and stimulated as indicated at *t* = 50 s and measurements acquired for a total of 180 s. Calcium levels were detected every 500 ms using excitation-emission ratios of 340/380 nm. Following each experiment *R*_max⁡_ and *R*_min⁡_ values were determined by the addition of 1% Triton-X100 and subsequent addition of 20 mM EGTA pH 7.4, respectively. 

### 2.8. Cytokine and Prostaglandin D_2_ (PGD2) Release

ELISAs for IL-6, IL-13, MCP-1, and TNF*α* (limits of detection 4 pg/mL, 4 pg/mL, 15 pg/mL, and 8 pg/mL resp.; eBioscience) and PGD2 (Cayman Chemicals) were performed on triplicate samples according to the suppliers' recommendations and developed using TMB substrate and absorbances were determined using a TECAN Sunrise Microplate Reader. 

### 2.9. Mast Cell Degranulation

The level of degranulation was determined using a modified colorimetric assay to assess the release of *β*-hexosaminidase. Mast cells (0.2 × 10^6^) were suspended in 200 *μ*L Tyrode's buffer supplemented with 1% FCS and stimuli were added for 30 min at 37°C. Reactions were terminated by centrifugation (400 g) and 50 *μ*L aliquots of supernatants assayed for release of *β*-hexosaminidase and normalised to total cellular *β*-hexosaminidase following cell lysis by the addition of 1% Triton-X 100 and by incubation with 1 mM *p*-nitrophenyl-*N*-acetyl-*β*-d-glucosamine (NAG) in 200 *μ*L 0.05 M citrate buffer, pH 4.5. After incubation in the dark at 37°C for 1 h the reaction was quenched by the removal of 62.5 *μ*L of the reaction mix into a clean well and the addition of 125 *μ*L/well 0.1 M sodium bicarbonate buffer and optical density determined by a TECAN Sunrise Microplate Reader at 405 nm. 

### 2.10. Western Blotting

Mast cells (2 × 10^6^/mL) were stimulated as indicated and reactions terminated by the addition of ice-cold PBS and centrifugation at 400 g at 4°C for 5 min. Lysis was performed by the addition of 50 *μ*L ice-cold, modified RIPA lysis buffer (50 mM Tris buffer, pH 7.4 containing 150 mM sodium chloride, 2% (v/v) NP40, 0.25% (w/v) sodium deoxycholate, 1 mM EGTA, 10 mM sodium orthovanadate, 0.5 mM phenylmethylsulfonylfluoride, chymostatin (10 *μ*g/mL), leupeptin (10 *μ*g/mL), antipain (10 *μ*g/mL), and pepstatin A (10 *μ*g/mL)). After vortexing, the cells were incubated on ice for 30 min before microcentrifugation of lysates at 12000 rpm for 15 min. The resulting supernatants (cell lysates) were stored at −20°C.

Equal protein loadings of cell lysates (30–40 *μ*g protein per lane), determined by BCA protein assay (bicinchoninic acid assay; Thermo Pierce), were resolved using the XCell *SureLock* Mini-Cell kit with NuPAGE Novex high-performance precast Bis-Tris gels and NuPAGE buffers and reagents (Invitrogen). Proteins were then transferred to nitrocellulose (Amersham) or PVDF membranes and protein loading was validated by Ponceau Red staining. Membranes were washed in Tris-buffered saline (TBS) (0.5 M NaCl and 20 mM Tris pH 7.5) with 0.1% (v/v) Tween-20 (TBS/Tween) and blocked for 1 h in TBS/Tween with 5% nonfat milk (Marvel). Membranes were then incubated overnight at 4°C with the appropriate primary detection antibody. All antibodies were diluted in TBS/Tween with either 5% nonfat milk or 5% BSA. Following incubation with primary antibody the membranes were washed with TBS/Tween and incubated in the appropriate horseradish peroxidase (HRP)-conjugated secondary antibody for 2 h at room temperature before visualisation using the ECL detection system and Kodak X-Ray film. Densitometry was performed using Image J software.

### 2.11. Statistical Analysis

All statistical analysis was performed using GraphPad Prism 5.0 (GraphPad Software Inc). Statistical analysis was by unpaired *t*-test or one-way ANOVA with Tukey's post-test and *P* is significant at *P < 0.05, **P < 0.01, ***P < 0.001, and ****P < 0.0001.

## 3. Results and Discussion

### 3.1. Phenotyping of PDMC, Mucosal-Type BMMC, and CTMC Mast Cell Subsets

**Figure 1:**
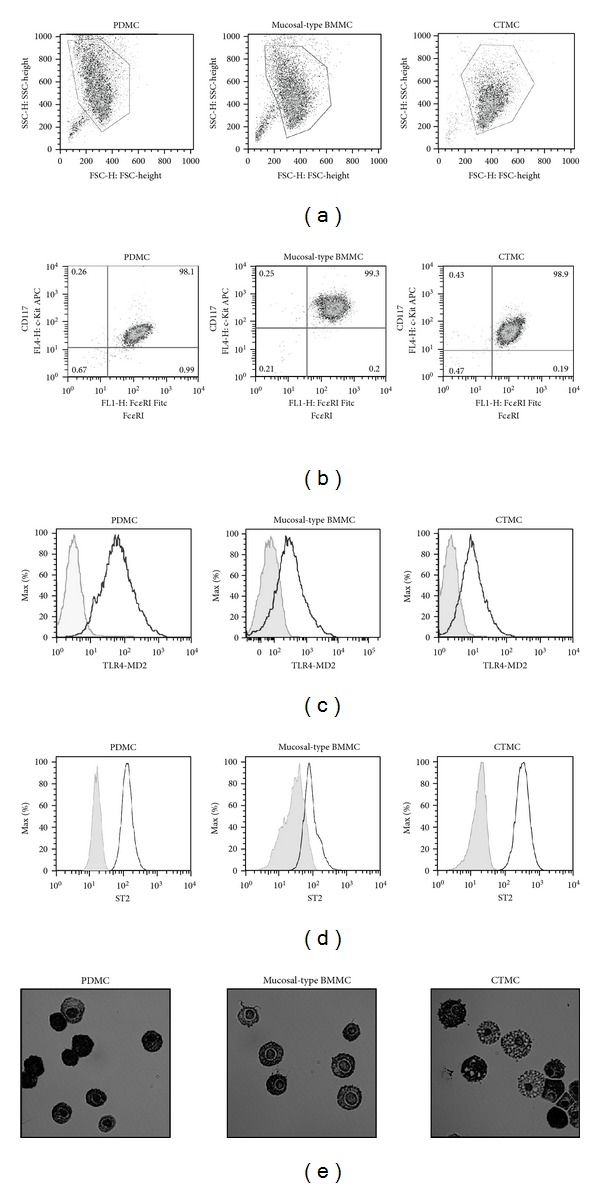
Phenotyping of mast cell subsets. Exemplar plots of mast cell phenotyping by flow cytometric analysis are shown in (a)–(d). FSC and SSC parameters of PDMC, mucosal-type BMMC, and CTMC cultured *in vitro* for 28 days (a) and gating (relative to isotype controls; not shown) of the consequent CD117^+^Fc*ε*R1^+^cell population (>98%; (b)) prior to the analysis of their TLR4^+^expression (c) are shown. In parallel experiments, ST2 expression of CD117^+^Fc*ε*R1^+^cells in the various populations was determined (d). Gray shaded plots (c-d) are isotype controls. In (e), exemplar images of toluidine blue staining of the mast cell populations (x10) are shown. The data are representative of at least 2 independent experiments.

Following expansion of PDMC and derivation of mucosal-type BMMC and CTMC for 4–6 weeks *in vitro*, these mast cell populations were phenotyped for mast cell lineage markers (CD117, Fc*ε*RI, and ST2) and also for TLR4 (toll-like receptor-4) and proteoglycan (heparin) expression (Figure 1). This analysis revealed all three mouse subsets to be of similar size, although the CTMC appeared rather less granular (Figure 1(a)), with each subset comprising a homogeneous population of CD117^+^Fc*ε*RI^+^ mast cells (Figure 1(b)). Moreover, all 3 populations expressed TLR4 (Figure 1(c)) and ST2 (Figure 1(d)), with BMMC typically expressing the highest levels of TLR4 and CTMC showing most ST2 expression. Similarly, PDMC, mucosal-type BMMC, and CTMC all contained heparin-containing granules as indicated by toluidine blue staining (Figure 1(e)), although there was some heterogeneity in the CTMC, and to a lesser extent, the mucosal-type BMMC populations, perhaps reflecting their differences in granularity (Figure 1(a)). Mucosal-type mast cells from mice have generally been considered to express little or no heparin, but in agreement with our results, it has been reported that heparin expression can be upregulated in response to SCF in these cells [14]: this functional plasticity is consistent with the ability of mouse BMMC to repopulate both serosal and mucosal compartments of mast cells *in vivo* [14]. 

### 3.2. Differential Functional Responses of Mast Cell Subsets

It has previously been reported that serosal- and mucosal-type mast cells exhibit differential functional responses with PDMC displaying strong degranulation responses whilst BMMC preferentially produce chemokines and cytokines, and that these responses can be further “fine-tuned” selectively in response to inflammatory stimuli and microenvironment [14]. We therefore characterised the differential responses of PDMC, mucosal-type BMMC, and CTMC in response to Ag-mediated crosslinking of Fc*ε*RI, LPS/TLR4 signalling and also the pharmacological stimulus PMA plus ionomycin (P/I) in terms of degranulation (*β*-hexosaminidase), eicosanoid (PGD2), chemokine (MCP-1), and cytokine (IL-6, IL-13 and TNF*α*) release (Table 1). These data confirmed that PDMC were the subtype that degranulated most strongly in response to Fc*ε*RI and PMA plus ionomycin and demonstrated that all of these subtypes generated *in vitro* exhibited little or no degranulation in response to LPS. All of the mast cell populations constitutively secreted high levels of PGD2 (lowest in PDMC); however, whilst PDMC did not produce any more PGD2 in response to Fc*ε*RI crosslinking, both mucosal-type BMMC and CTMC responded further to this stimulus and also to LPS. Further differential responses were observed in terms of chemokine release as whilst LPS stimulated release of MCP-1 in all subtypes, Fc*ε*RI crosslinking induced little or no release of MCP-1 over the basal levels in PDMC, yet strongly stimulated release of this chemokine from mucosal-type BMMC and CTMC. Only PMA plus ionomycin were able to induce substantial secretion of IL-6 and IL-13 by PDMC, and none of the stimuli were routinely able to trigger TNF*α* release by these cells. Similarly, whilst Fc*ε*RI- and LPS/TLR4-signalling induced little or no IL-13 or TNF*α* production by CTMC, LPS, but not Fc*ε*RI crosslinking, triggered strong secretion of IL-6. By contrast, Fc*ε*RI and LPS/TLR4 signalling both induced the production of all three cytokines (IL-6, IL-13 and TNF*α*) by mucosal-type BMMC. It has been reported that BMMC and freshly isolated PDMC derived from C57BL/6 mice exhibited higher levels of degranulation (*β*-hexosaminidase) and generated lower levels of cytokine and prostaglandin production than those derived from BALB/c mice [38] but we did not find this to be a significantly reproducible trend in this study (data are not shown).

### 3.3. ES-62 Inhibits Functional Responses in Both Serosal and Mucosal Mast Cell Subtypes

**Figure 2:**
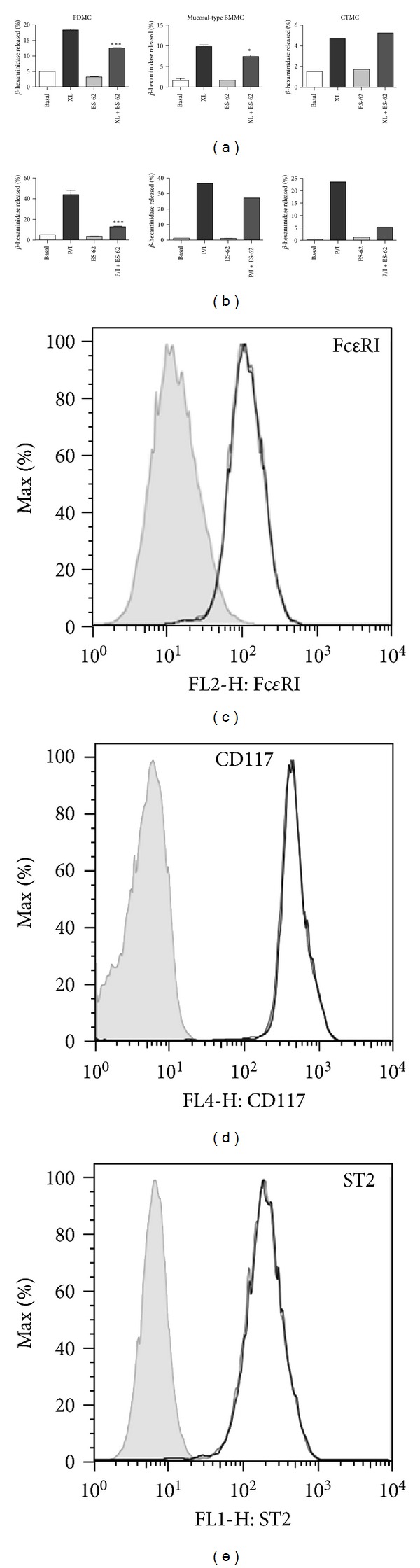
Degranulation by mast cell subsets. Mucosal-type BMMC, CTMC and PDMC were sensitised with murine anti-DNP IgE (0.5 *μ*g/mL) in the presence and absence of ES-62 (2 *μ*g/mL) overnight. Cells were then stimulated with DNP (0.5 *μ*g/mL) to induce Fc*ε*RI cross-linking (XL; (a)) or PMA plus ionomycin (both 1 *μ*M; (b)) for 30 min at 37°C. Degranulation was determined as the %  *β*-hexosaminidase release relative to the total enzyme activity of the cells and the data presented are from single experiments representative of at least 2 independent experiments. PDMC were sensitised with murine anti-DNP IgE (0.5 *μ*g/mL) in the presence and absence of ES-62 (2 *μ*g/mL) overnight and analysed for expression of Fc*ε*RI (c), CD117 (d), and ST2 (e). Grey shaded plots (c–e) are relevant isotype controls.

Given the differential functional responses of the mast cell subtypes, we next investigated whether the filarial nematode product showed selectivity in its desensitisation of mast cell responses, both in terms of the mast cell subtype targeted and also with respect to their differential responses to the individual proinflammatory stimuli. These studies showed that exposure to ES-62 significantly inhibited the degranulation of PDMC and the mucosal-type BMMC, but not CTMC, in response to Fc*ε*RI signalling (Figure 2(a)). Whilst the responses to LPS were typically too low (Table 1) to show significant effects of ES-62 (data not shown), degranulation in response to PMA plus ionomycin was also significantly inhibited in PDMC (Figure 2(b)) and likewise observed in CTMC (2/2 experiments) and the mucosal-type BMMC (3/5 experiments). 

**Figure 3:**
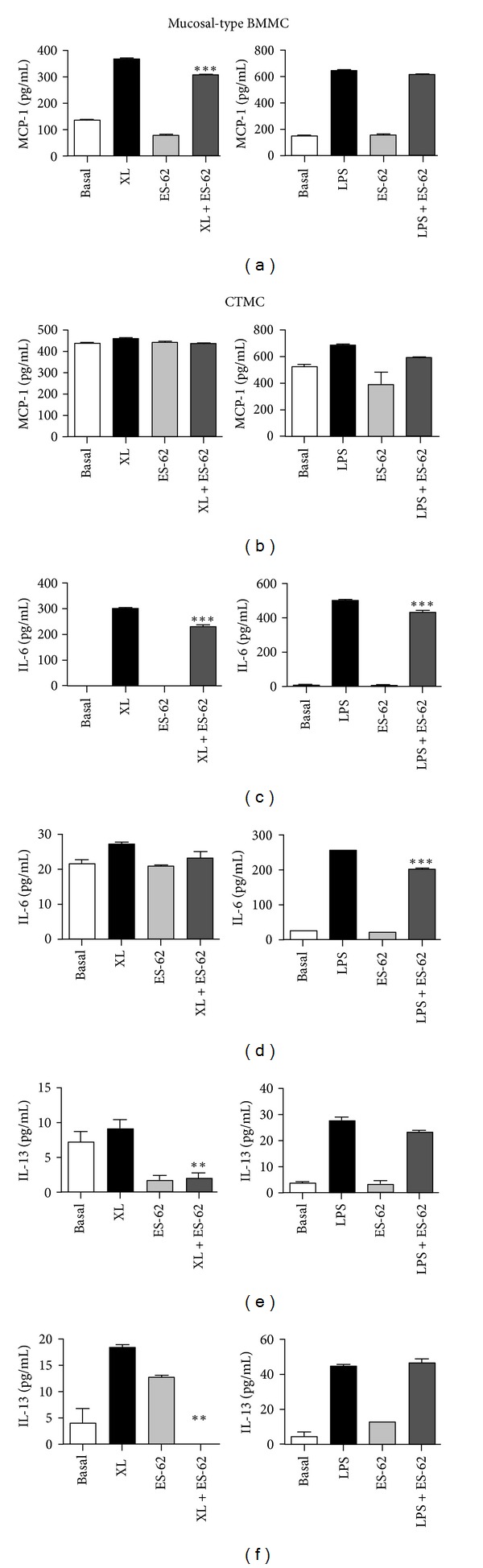
Chemokine and cytokine release by mast cell subsets. Mucosal-type BMMC (a), (c) & (e) and CTMC (b), (d) & (f) were sensitised with murine anti-DNP IgE (0.5 *μ*g/mL) in the presence and absence of ES-62 (2 *μ*g/mL) overnight. Cells were then stimulated with DNP (0.5 *μ*g/mL) to induce Fc*ε*RI cross-linking (XL) or LPS (0.5 *μ*g/mL) for 24 h at 37°C and MCP-1 (a) & (b), IL-6 (c) & (d), and IL-13 (e) & (f) release measured by ELISA. The data presented are single experiments representative of at least 2 independent experiments apart from the IL-13 release from CTMC, which could only be detected in a single experiment.

Although PDMC produced little or no chemokines/cytokines in response to either Fc*ε*RI crosslinking or LPS-stimulation (Table 1), generally, the very low levels of MCP-1, IL-6, and IL-13 observed were inhibited by ES-62 (data not shown). With respect to mucosal-type BMMC and CTMC, whilst ES-62 was only able to significantly inhibit MCP-1 production by Fc*ε*RI-stimulated mucosal-type BMMC (Figures 3(a) and 3(b)), it inhibited LPS-stimulated IL-6 production by both subtypes as well as that seen in mucosal-type BMMC in response to crosslinking of Fc*ε*RI (Figures 3(c) and 3(d)). By contrast, the IL-13 response to Fc*ε*RI crosslinking was suppressed by ES-62 in both subtypes (Figures 3(e) and 3(f)) whilst the Fc*ε*RI-mediated TNF*α* response only seen in mucosal-type BMMC was also inhibited by ES-62 (data not shown). 

Collectively, therefore, we have shown that ES-62 can target both serosal- and connective-tissue phenotypes to render these cells hyporesponsive to proinflammatory stimuli. That ES-62 can modulate the responses of both mature and immature cells is consistent with our previous studies showing that the parasite product can target immature bone marrow progenitors of macrophages and dendritic cells to generate a more anti-inflammatory environment *in vivo* [37, 39]. The finding that ES-62 did not modulate expression of Fc*ε*RI, CD117, or ST2 on resting or sensitized mast cells (Figures 2(c)–2(e) and data not shown), however, suggested that it was not affecting their phenotypic status but rather targeting their functional plasticity. The observed differential targeting of particular responses may indicate selective actions in particular microenvironments and consequently, recruitment of other innate cells such as neutrophils to the site of inflammation as well as mast cell promotion of the polarisation of particular immune responses [4, 14], dependent on the site and type of inflammation (e.g., protective inflammation to fight infection versus aberrant autoimmune or allergic hyperinflammation). 

### 3.4. ES-62 Targets Calcium and PKC Signalling in Mast Cells

To address how ES-62 may be differentially targeting the functional responses of PDMC, the mucosal-type BMMC, and CTMC, we investigated the effects of the parasite product on calcium mobilisation and expression of PKC*α*, as we had previously shown modulation of these two key signals in degranulation and cytokine signalling [40–42] to be crucial to the desensitisation of Fc*ε*RI-mediated human mast cell responses [35, 36]. Moreover, ES-62 exerts its effects via subversion of TLR4 signalling whilst the canonical TLR4 ligand LPS typically acts to enhance Fc*ε*RI functional responses [43, 44], the latter accounting at least in part for the widely established finding that LPS exacerbates airway hyperresponsiveness [43]. LPS has been reported to do this by increasing Fc*ε*RI-driven calcium mobilisation by upregulating the Orai1 and Stim1 subunits of the store-operated calcium (SOC) channel and hence stimulating calcium influx [43]. In addition, PKC signalling, including that of PKC*α*, has been shown to be important to LPS/TLR4 responses in a variety of innate cells [45]. 

**Figure 4:**
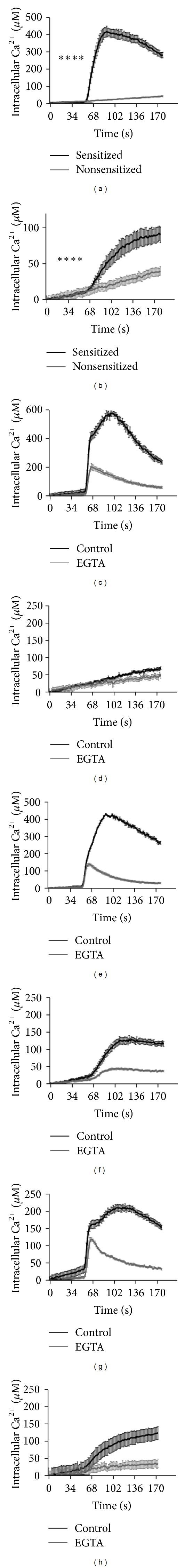
Calcium mobilisation in mast cell subsets. Fura-2/AM-loaded resting, nonsensitised (a) & (b) or anti-DNP IgE (0.5 *μ*g/mL)-sensitised (a)–(h) mucosal-type BMMC (e) & (f), CTMC (g) & (h) and PDMC (a)–(d) were stimulated at 50 s with DNP (0.5 *μ*g/mL) to induce cross-linking (XL) of Fc*ε*R1 (a), (c), (e) & (g) or 0.5 *μ*g/mL LPS (b), (d), (f) & (h), and intracellular calcium mobilisation and influx recorded in real time using excitation-emission ratios of 340/380 nm (a)–(h). For analysis of intracellular mobilisation alone, the cells were stimulated in calcium free HBSS supplemented with 100 *μ*M EGTA to remove all extracellular calcium (EGTA). Calcium levels were calculated from *R*_max⁡_ and *R*_min⁡_ values and the data are presented as the mean calcium values of triplicate samples (base line calcium values subtracted) from a single experiment representative of at least 3 independent experiments.

**Figure 5:**
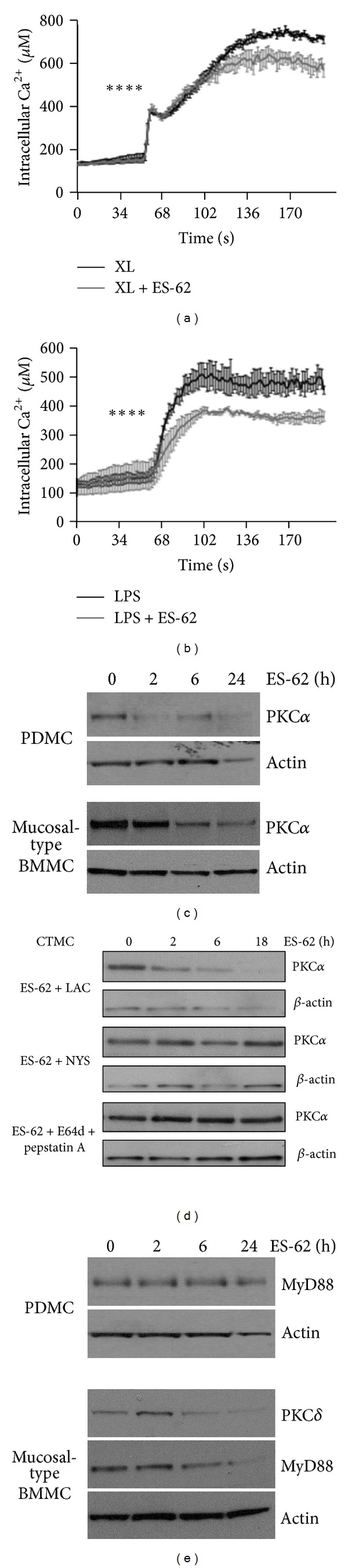
ES-62 modulates signalling in mast cell subsets. PDMC were sensitised with murine anti-DNP IgE (0.5 *μ*g/mL) in the presence or absence of ES-62 (2 *μ*g/mL) overnight. Following loading with Fura-2/AM such PDMC were stimulated at 50 s with DNP (0.5 *μ*g/mL) to induce cross-linking (XL) of Fc*ε*R1 (a) or 0.5 *μ*g/mL LPS (b) and intracellular calcium mobilisation and influx recorded in real time using excitation-emission ratios of 340/380 nm (a) & (b). Calcium levels were calculated from *R*_max⁡_ and *R*_min⁡_ values and data are presented as the mean calcium values of triplicate samples from a single experiment representative of at least 3 independent experiments. PDMC and mucosal-type BMMC (c) & (e) were cultured with ES-62 (2 *μ*g/mL) for the indicated times and expression of PKC*α* ((c), Cell Signalling Technology), MyD88 ((e), Abcam) and PKC*δ* ((e), Cell Signalling Technology) analysed by Western Blotting. In (d), following preincubation for 1 h with inhibitors of proteosomal degradation (10 *μ*M Lactacystin, ENZO Life Sciences; LAC), caveolae/lipid raft trafficking (50 *μ*g/mL Nystatin; NYS) and lysosomal degradation (E64d + pepstatin A both 10 *μ*g/mL, ENZO Life Sciences), sensitised CTMC were cultured with ES-62 (2 *μ*g/mL) for the indicated times and expression of PKC*α* analysed by Western Blotting. Actin was used as a loading control and ES-62-mediated downregulation of PKC*α* expression was observed in PDMC, Mucosal-type BMMC, and CTMC in at least 2 independent experiments.

As a first step, we investigated whether Fc*ε*RI- and LPS/TLR-signalling induced calcium mobilisation in each of PDMC, mucosal-type BMMC and CTMC (Figure 4). Interestingly we found that not only as expected, IgE-sensitisation of mast cells was essential for Fc*ε*RI-mediated calcium mobilisation, but also that it enhanced that seen in response to LPS (PDMC; Figures 4(a) and 4(b)). Moreover, it was clear that whilst the Fc*ε*RI signal reflected a mix of mobilisation of intracellular calcium and calcium influx, as indicated by the observed transient spike in the absence of extracellular calcium (EGTA), the calcium response to LPS in all mast cell subtypes predominantly reflected calcium influx (Figures 4(c)–4(h)). Consistent with LPS inducing calcium influx, we also found as reported previously [43] that LPS enhanced Fc*ε*RI-mediated calcium mobilisation (data not shown). By contrast, although preexposure to ES-62 did not modulate the baseline calcium levels, the parasite product suppressed the subsequent calcium mobilisation in response to both Fc*ε*RI-crosslinking and LPS/TLR4 signalling in PDMC (Figures 5(a) and 5(b)).

In addition, ES-62 was found to downregulate PKC*α* expression in PDMC, mucosal-type BMMC and CTMC (Figures 5(c) and 5(d)), suggesting, that as with human mast cells [35], ES-62 was targeting this signal in PDMC, mucosal-type BMMC and CTMC to suppress degranulation and cytokine responses. PKC*α* has been shown to be degraded via both proteosomal and caveolae/lipid raft, lysosomal routes [46, 47] and our preliminary studies in human mast cells showed that the inhibitor of caveolae/lipid raft trafficking, nystatin could protect against such downregulation following exposure to ES-62 for 24 h [35]. Furthermore, our earlier studies [48] had shown that ES-62-mediated downregulation of PKC*α* expression in B cells could be prevented by treatment with the cysteine protease inhibitor leupeptin; findings also consistent with a lysosomal mechanism [49, 50] of degradation of this key signalling element. We have therefore further investigated the (differential) mechanisms involved in ES-62-driven degradation of PKC*α* in mast cell subtypes. Our data in CTMC are consistent with that of our previous study on human mast cells [35] as they showed that nystatin but not lactacystin protected against PKC*α* degradation and we have further confirmed the role of an endosomal route by showing that protection is also afforded by the combination of the lysosomal inhibitors, E64d plus pepstatin A (Figure 5(d)). However, our comprehensive analysis of mechanism in PDMC and the mucosal-type BMMC has revealed a more complicated scenario in which both nystatin and lactacystin can offer some protection at differential time points (data not shown). Interestingly, these findings are consistent with reports that both mechanisms can coexist in cells not only in a temporal and spatially distinct manner but can also be triggered to regulate PKC*α* expression in response to a single agonist [46]. Hence, the differential recruitment of one or more of these degradative pathways may provide a rationale for fine tuning the level of PKC*α* desensitisation required to downregulate hyperinflammatory responses; an attractive proposal gave that CTMC exhibit the least degranulation potential and are not as effective at producing cytokines as the mucosal-type BMMC. Overall, as we have also found that the inhibitors alone can modulate PKC*α* expression in PDMC and mucosal-type BMMC, these findings collectively indicate that regulation of this key signalling element in mast cells is tightly controlled by a complex and dynamic system involving both proteosomal and lysosomal routes of degradation.

Finally, whilst we have shown that ES-62 can suppress the cytokine responses of both PDMC and the mucosal-type BMMC, it is clear that the levels of cytokines produced by PDMC in response to Fc*ε*RI- and LPS/TLR4 signalling are very low compared to those secreted by mucosal-type BMMC. We have therefore addressed identifying which signals may be contributing to such higher levels of cytokine production by also determining the effects of ES-62 on PKC*δ* expression as this signalling element has not only been shown to be important for functional responses to Fc*ε*RI- and LPS/TLR4 signalling [40, 45, 51–53] but also to be a target for downregulation by the parasite product in human mast cells and B cells [35, 48]. In addition, we have also examined the effect of ES-62 on MyD88, a pivotal signal transducer of TLR4 [45, 51, 54] as we have shown it to be a target of ES-62 in countering Th17 pathology [37] and only the MyD88-, and not the TRIF-dependent pathway of TLR4 signalling, appears to be active in BMMC [55]. Consistent with the hypothesis that additional signals such as MyD88 and PKC*δ* are required for the augmented cytokine responses observed in BMMC relative to PDMC, these studies show that whilst MyD88 expression in PDMC is unchanged by exposure to ES-62, culture with the parasite product results in downregulation of both MyD88 and PKC*δ* in mucosal-type BMMC (Figure 5(e)).

## 4. Conclusions

PDMC, mucosal-type BMMC, and CTMC mast cell populations display differential functional responses with mature serosal mast cells predominantly acting like cells that perform a specialised degranulation function. By contrast, BMMC, which have been reported to possess an immature mucosal-like phenotype that can further differentiate into mucosal or serosal mast cells display reduced degranulation and increased cytokine responses. Consistent with the idea that BMMC are plastic and can differentiate into either mucosal or serosal mast cells, CTMC display a comparable degranulation potential to that of mucosal-type BMMC and a cytokine profile intermediate of mucosal-type BMMC and mature serosal/connective tissue PDMC. All three mast cell populations can be rendered hyporesponsive by ES-62 but the selective nature of these effects suggests that ES-62 may be targeting functions of the individual subtypes that are specific to the particular inflammatory microenvironment and phenotype. 

**Figure 6:**
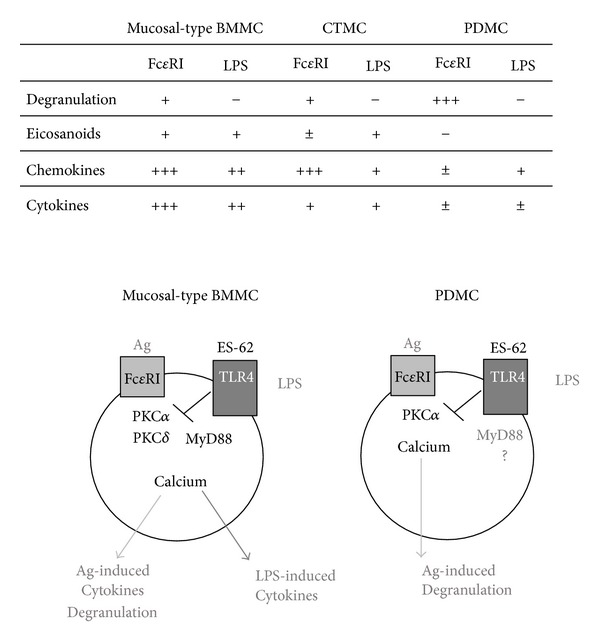
Model of differential desensitisation of mast cell subsets by ES-62. Mucosal-type BMMC, CTMC, and PDMC exhibit differential functional responses to antigen (Ag)-mediated cross-linking of Fc*ε*RI and stimulation with LPS as summarised. The signalling mechanisms underlying the coupling to these differential responses and their desensitisation by ES-62 have not been fully delineated but our working model is that the Fc*ε*RI-mediated degranulation observed in PDMC involves PKC*α* and calcium mobilisation which are effectively targeted by ES-62. Although these cells express TLR4/MyD88, they are uncoupled from downstream functional LPS signalling by an as yet unknown mechanism. By contrast, mucosal-type BMMC exhibit both degranulation (via Ag/Fc*ε*RI) and cytokine (via Fc*ε*RI and LPS/TLR4) responses, requiring recruitment of additional signals such as PKC*δ* and MyD88 signalling which are both also targeted by ES-62.

The mechanisms underlying such desensitisation have not been fully delineated but our working model (Figure 6) is that reduced degranulation and low level cytokine secretion reflect desensitisation of Fc*ε*RI- and LPS/TLR4-mediated calcium mobilisation and PKC*α* signalling whilst suppression of the high levels of cytokine production by mucosal-type BMMC in response to these signals requires downregulation of additional signals such as MyD88 and PKC*δ*. Such a rheostat effect allowing differential signal strength-dependent desensitisation of receptor signalling would allow ES-62 to provide an appropriate level of hyporesponsiveness that would prevent development of aberrant autoimmune and allergic inflammatory disorders whilst allowing appropriate levels of inflammation to generate protective immune responses to pathogenic infection.

## Figures and Tables

**Table 1:** Differential functional responses of mast cell subsets.

	PDMC				Mucosal-type BMMC				CTMC			
	Basal	Fc*ε*RI	LPS	P/I	Basal	Fc*ε*RI	LPS	P/I	Basal	Fc*ε*RI	LPS	P/I
Degranulation (%)	4.48 ± 0.56	33.24 ± 4.07	2.87 ± 1.47	55.34 ± 4.24	7.46 ± 1.10	17.34 ± 2.19	2.87 ± 1.78	31.97 ± 3.78	3.95 ± 1.02	17.3 ± 2.90	2.173 ± 0.48	25.29 ± 4.62
		*n* = 7	*n* = 2	*n* = 4		*n* = 5	*n* = 3	*n* = 5		*n* = 2	*n* = 2	*n* = 2

PGD2 (pg/mL)	1804 ± 44	1907 ± 55			3257 ± 338	4711 ± 264	4509 ± 156		2888 ± 370	3229 ± 333	4764 ± 153	
		*n* = 3				*n* = 5	*n* = 5			*n* = 5	*n* = 2	

IL-6 (pg/mL)	70 ± 27	67 ± 32	81.33 ± 7	408 ± 61	37.4 ± 14.7	560 ± 96	2557 ± 696	21976 ± 6589	87 ± 23	114 ± 19	812 ± 217	
		*n* = 6	*n* = 4	*n* = 3		*n* = 18	*n* = 18	*n* = 4		*n* = 3	*n* = 4	

IL-13 (pg/mL)	7.81 ± 1.52	17.91 ± 2.84	25.91 ± 4.43	248 ± 20	5.88 ± 0.86	366 ± 88	338 ± 116	1578 ± 111	3.94 ± 2.94	18.5 ± 0.45	44.7 ± 1.1	
		*n* = 6	*n* = 7	*n* = 3		*n* = 6	*n* = 5	*n* = 3		*n* = 1	*n* = 1	

MCP-1 (pg/mL)	457 ± 73	387 ± 84	616 ± 109	102 ± 19	504 ± 212	1703 ± 393	862 ± 296		396 ± 58	1410 ± 226	511 ± 76	
			*n* = 2			*n* = 2	*n* = 3			*n* = 2	*n* = 2	

TNF*α*	15.25 ± 2.48	15.57 ± 2.91	19.62 ± 3.38	32.33 ± 2.94	5.02 ± 2.12	506 ± 159	150 ± 38	3199 ± 916	1.42 ± 0.19	1.41 ± 0.34	15.62 ± 1.61	
		*n* = 1	*n* = 1			*n* = 12	*n* = 9	*n* = 4		*n* = 1	*n* = 2	

Data are presented as the mean values ± SEM where *n* = number of independent experiments or mean values ± SD of triplicate samples in the case where data from a single experiment are presented.
